# Eel Kisspeptins: Identification, Functional Activity, and Inhibition on both Pituitary LH and GnRH Receptor Expression

**DOI:** 10.3389/fendo.2017.00353

**Published:** 2018-01-08

**Authors:** Jérémy Pasquier, Anne-Gaëlle Lafont, Florian Denis, Benjamin Lefranc, Christophe Dubessy, Antonio Moreno-Herrera, Hubert Vaudry, Jérôme Leprince, Sylvie Dufour, Karine Rousseau

**Affiliations:** ^1^Muséum National d’Histoire Naturelle, Research Unit BOREA, Biology of Aquatic Organisms and Ecosystems, CNRS 7208, IRD207, UPMC, UCN, UA, Paris, France; ^2^Laboratory of Neuronal and Neuroendocrine Differentiation and Communication, INSERM U1239, Normandy University, Rouen, France; ^3^Department of Cell Biology, Physiology, and Immunology, Maimonides Institute for Biomedical Research of Cordoba (IMIBIC), Reina Sofia University Hospital, University of Córdoba, Córdoba, Spain

**Keywords:** kisspeptins, tissue distribution, functional activity, pituitary cell culture, LH, GnRH receptor, European eel

## Abstract

The European eel (*Anguilla anguilla*) presents a blockade of sexual maturation at a prepubertal stage due to a deficient production of gonadotropins. We previously initiated, in the eel, the investigation of the kisspeptin system, one of the major gatekeepers of puberty in mammals, and we predicted the sequence of two *Kiss* genes. In the present study, we cloned and sequenced *Kiss1* and *Kiss2* cDNAs from the eel brain. The tissue distributions of *Kiss1* and *Kiss2* transcripts, as investigated by quantitative real-time PCR, showed that both genes are primarily expressed in the eel brain and pituitary. The two 10-residue long sequences characteristic of kisspeptin, eel Kp1(10) and Kp2(10), as well as two longer sequences, predicted as mature peptides, eel Kp1(15) and Kp2(12), were synthesized and functionally analyzed. Using rat Kiss1 receptor-transfected Chinese hamster ovary cells, we found that the four synthesized eel peptides were able to induce [Ca^2+^]_i_ responses, indicating their ability to bind mammalian KissR-1 and to activate second messenger pathways. In primary culture of eel pituitary cells, all four peptides were able to specifically and dose-dependently inhibit *lhβ* expression, without any effect on *fshβ*, confirming our previous data with heterologous kisspeptins. Furthermore, in this eel *in vitro* system, all four peptides inhibited the expression of the type 2 GnRH receptor (*gnrh-r2*). Our data revealed a dual inhibitory effect of homologous kisspeptins on both pituitary *lhβ* and *gnrh-r2* expression in the European eel.

## Introduction

The European eel, *Anguilla anguilla*, exhibits a complex life cycle, with a blockade of sexual maturation at a prepubertal stage (silver stage) as long as its oceanic reproductive migration is prevented. This blockade is due to a deficient production of pituitary gonadotropins (1). The understanding of the mechanisms regulating the gonadotropic axis and controlling eel reproduction is of particular interest, considering the drastic decline of wild populations (2) and the current lack of self-sustained aquaculture of this species. Furthermore, as the eel is a representative species of an early group of teleosts (the elopomorpha) (3), deciphering such mechanisms in this species may provide new insights on evolution and ancestral regulations of endocrine systems.

Kisspeptin (*Kiss1*) gene was first discovered as a metastasis suppressor gene in human melanoma (4). Soon after, an orphan receptor, GPR54, was cloned in the rat (5) and was subsequently identified as the cognate receptor of kisspeptins (Kiss), the natural peptides derived from the product of *Kiss1* (6–8). In 2003, a major breakthrough in reproductive endocrinology was achieved, as the GPR54 gene was shown to be essential for the onset of puberty. Mutations in *gpr54* caused hypogonadotropic hypogonadism in humans (9, 10) and mice (10, 11). Later, hypogonadotropic hypogonadism was also observed in *Kiss1* knockout mice (12), while precocious puberty onset occurred in humans with either *gpr54*-activating mutation leading to prolonged *in vitro* activation of intracellular signaling pathways in response to kisspeptin (13) or *Kiss1* mutations leading to higher *in vitro* kisspeptin resistance to degradation (14). Since these discoveries, the kisspeptin system has been considered as a major puberty gatekeeper and reproductive regulator, upstream of GnRH [for reviews see Ref. (15–17)]. Nevertheless, Tang and collaborators (18) recently showed that gametogenesis and reproductive capability are not impaired in zebrafish mutant lines for Kiss, as well as for kisspeptin receptors (KissR), suggesting that the Kiss/KissR systems may be dispensable for the reproduction of some non-mammalian vertebrates.

The kisspeptin system has been identified in a number of vertebrate species, leading to the discovery of multiple genes encoding kisspeptins (from *Kiss1* to *Kiss3*) as well as multiple genes encoding its receptors (from *KissR-1* to *KissR-4*) [for review see Ref. (19)]. To date, mature amidated kisspeptins have been purified only from human, *Xenopus*, turtle, and salmon. In human, multiple mature Kiss1 peptides (Kp1), including a mature peptide encompassing 54-aa [Kp1(54)] and shorter peptides [Kp1(14) and Kp1(13)], were isolated from placental extracts (6). Mature Kiss peptides, cleaved from the same precursor, share the same C-terminal 10-aa sequence, which is the minimal sequence required to specifically bind their cognate receptor, as first described for human Kiss1 by Kotani et al. (6). Although *Kiss2-like* gene has been identified in human, a Kiss2 peptide (Kp2) may not be produced as an endogenous ligand due to the lack of an amidation signal in the precursor polypeptide (20). In *Xenopus laevis*, a species presenting three *Kiss* genes (*Kiss1, 2*, and *3*), only Kp2(12) has been isolated by HPLC (21). In the red-eared slider turtle (*Trachemys scripta*), the mature endogenous Kiss2 peptide is a 12-aa sequence, while in the masu salmon (*Oncorhynchus masou*), it is a 13-aa sequence (20).

In the eel, three Kiss receptor genes have been characterized, i.e., *KissR-1, KissR-2*, and *KissR-3* (19, 22–24). Their expressions mainly occur in the brain, pituitary, and gonads (19). Using heterologous Kiss peptides [human/lamprey Kp1(10); human Kp1(14); lamprey Kp1(13); and zebrafish Kp1(10), 2(10), 1(15), and 2(15)], we previously showed an unexpected and specific *in vitro* inhibitory effect on luteinizing hormone (*lh*β) expression by eel pituitary cells in primary culture (22). Three GnRH receptors were identified in the eel, two of type I (*Gnrh-r1a and 1b*) and one of type II (*Gnrh-r2*); all were shown to be expressed in the brain, pituitary, and gonads (25).

In the present study, we report the cloning of two eel Kiss transcripts (*Kiss1* and *Kiss2*), corresponding to the two previously defined ORFs (23). Using a specific quantitative real-time PCR (qPCR) approach, we investigated their distributions in various eel tissues. We synthesized the two minimal 10-aa peptides, eel Kp1(10) and Kp2(10), as well as the predicted mature peptides, eel Kp1(15) and Kp2(12). We tested all four peptides on rat KissR-1-transfected Chinese hamster ovary (CHO-K1) cells. We also studied their biological effects on the expression of gonadotropins (*lh*β; follicle-stimulating hormone, *fsh*β) and gonadotropin-releasing hormone receptors (*gnrh-r*) by eel pituitary cells in primary culture.

## Materials and Methods

### Animals

European female eels were at the prepubertal “silver” stage, which corresponds to the last continental phase of the eel life cycle, preceding the oceanic reproductive migration. Eels were purchased from Gebr. Dil import-export BV (Akersloot, The Netherlands) and transferred to MNHN, France.

Animals were anesthetized by cold and then killed by decapitation under the supervision of authorized person (Karine Rousseau; No. R-75UPMC-F1-08) according to the protocol approved by Cuvier Ethic Committed (No. 68-027).

### Cloning and Sequencing of European Eel *Kiss1* and *Kiss2* cDNAs

Total RNA from eel brain (pooled di-/mesencephalon) was extracted using Trizol reagent and reverse transcribed as previously described (22).

Predicted genomic sequences of eel *Kiss1* and *Kiss2* (23, 26) were used to design specific *Kiss1* and *Kiss2* primers, respectively (Table 1). Using the Advantage 2 PCR Kit (Clontech Laboratories Inc., PaloAlto, CA, USA), RACE PCRs with 5′-cDNA or 3′-cDNA as templates were performed as follows: an initial step of polymerase activation for 3 min at 94°C; then 10 cycles of 30 s at 94°C for denaturing, 30 s at 70°C for annealing, 90 s at 72°C for primer extension; and then 25 cycles of 30 s at 94°C for denaturing, 30 s at 68°C for annealing, 90 s at 72°C for primer extension, and a single final extension step of 5 min at 72°C. PCR products of appropriate estimated size were sequenced at GATC Biotech Ltd. (Konstanz, Germany).

**Table 1:** Primers used in the 3′- and 5′-RACE PCR and quantitative real-time PCR (qPCR) amplifications.

Primers	5′–3′ sequence (bp)	
**Primers for 3′-RACE PCR**		
Kiss1-F	CGCCACAAGCGGCCAAGAAG	
Kiss2-F	AGGGCCACATTTCCTGCCGACT	
**Primers for 5′-RACE PCR**		
Kiss1-R	CCCGCTTCTTGGCCGCTTGT	
Kiss2-R	CCGAACGGATTGCGGTTGAATTTG	
**Primers for qPCR**		
*Kiss1*-F	GGTCTCTTAGGTACACCCCGT	This study
*Kiss1*-R	ACAGCTCCTCGCTCATTTG	
*Kiss2*-F	ACGGACGACTCAGGTTCTCT	This study
*Kiss2*-R	GCCCTCGATTTCACTGTCTT	
*actin*-F	AGTATTTGCGCTCGGGTG	Aroua et al. (27)
*actin*-R	CAGCCTTCCTTCCTGGGT	
*lhβ*-F	TCACCTCCTTGTTTCTGCTG	Aroua et al. (27)
*lhβ*-R	TAGCTTGGGTCCTTGGTGATG	
*fshβ*-F	TCTCGCCAACATCTCCATC	Aroua et al. (27)
*fshβ*-R	AGAATCCTGGGTGAAGCACA	
*gnrh-r1a*-F	TGGTCATGAGTTGCTGCTACA	Penaranda et al. (25)
*gnrh-r1a*-R	AGACACCCCTCTCCGTCTTT	
*gnrh-r1b*-F	TCGTCACGCTCTACGTTGTC	Penaranda et al. (25)
*gnrh-r1b*-R	AGGCAGGACTCTCCACCTTT	
*gnrh-r2*-F	TCACCTTCTCCTGCCTCTTC	Penaranda et al. (25)
*gnrh-r2*-R	TTGGAAGATGCCTTCCCTTT	

The signal peptides of the Kiss precursors were predicted using SignalP tool (28). Cleavage and amidation sites, as well as mature peptides, were predicted from the Kiss precursor using NeuroPred tool (29).

### Synthesis of Eel Kp1 and 2 Peptides

European eel Kp1(10), Kp2(10), Kp1(15), and Kp2(12) (Table 2) were synthesized (0.1-mmol scale) by the solid-phase methodology on a Rink amide 4-methylbenzhydrylamine resin (Biochem, Meudon, France) using a 433A peptide synthesizer (Applied Biosystems, Courtaboeuf, France) and the standard procedure, as previously described (30). The synthetic peptides were purified by reversed-phase (RP) HPLC on a 2.2 cm × 25 cm Vydac 218TP1022 C_18_ column (Alltech, Templemars, France), using a linear gradient (20–40% over 60 min) of acetonitrile/TFA (99.9:0.1, v/v) in water, at a flow rate of 10 ml/min. Analytical RP-HPLC, performed on a 0.46 cm × 25 cm Vydac 218TP54 C_18_ column, showed that the purity of the peptide was greater than 99%. The molecular mass of the peptide was verified by mass spectrometry on a MALDI-TOF Voyager DE-PRO instrument (Applied Biosystems).

**Table 2:** Sequences of predicted eel kisspeptins.

Peptide	Symbol	Sequence	Length (aa)
Eel 1 kisspeptin-10	Kp1(10)	YNWNSFGLRY-NH2	10
Eel 1 kisspeptin-15	Kp1(15)	ENFSSYNWNSFGLRY-NH2	15
Eel 2 kisspeptin-10	Kp2(10)	FNRNPFGLRF-NH2	10
Eel 2 kisspeptin-12	Kp2(12)	SKFNRNPFGLRF-NH2	12

### Functional Activity of Eel Kp1 and Kp2 Peptides in Rat KissR-1-Transfected CHO-K1 Cells

CHO-K1 cells were cultured to semiconfluence in 12-well plates using Ham’s F12 medium supplemented with 10% fetal bovine serum and 1% antibiotic–antimycotic solution. Cells were transfected with different quantities of the recombinant plasmid, ranging from 3 to 5 µg, using Lipofectamine 2000 (Invitrogen, Cergy Pontoise, France) as previously described (30). Twenty-four hours after transfection, media were replaced by fresh F12 medium containing 1 mg/ml G418 (Geneticin; Life Technologies, Inc.). One week later, surviving cells were detached, diluted, and plated on 96-well plates at 0.7 cells/well. Monoclonal cell lines expressing rat KissR-1 (CHO-K1-rKissR-1) were followed daily by contrast phase microscopy. Seven independent monoclonal stable cell lines were obtained after a 3-week period of selection. By using qPCR, two cell lines were selected based on their expression levels being closest to physiological levels among all cell lines. Of these, one cell line was definitely selected based on its best performance in showing clear Ca^2+^-mobilizing responses to treatment with rat Kp1(10).

To compare the KissR-1 agonistic activities of eel Kp1(10), Kp2(10), Kp1(15), and Kp2(12), the level of [Ca^2+^]_i_ after stimulation of CHO-K1 cells stably expressing KissR-1 by these peptides was monitored by spectrofluorometry as previously described (30, 31) with slight modifications. Briefly, after 24 h in culture, cells were incubated for 1 h in a humidified incubator (37°C, 5% CO_2_) with 2 µM Fluo-4 acetoxymethyl ester (AM) calcium dye (Life Technologies, Saint Aubin, France) in Hank’s Buffer Saline Solution (HBSS; Life Technologies) buffered with 5 mM HEPES and supplemented with 2.5 mM probenecid (Sigma-Aldrich, Saint-Quentin Fallavier, France). Cells were washed twice with HBSS/HEPES/probenecid to remove Fluo-4 AM from the incubation medium and incubated in 150 µl of the same medium at 37°C for 15 min. Fluorescence was recorded using a Flexstation 3 fluorescence plate reader system (Molecular Devices, Saint-Grégoire, France) during 180 s with an excitation wavelength of 480 nm, an emission wavelength of 525 nm, and a cutoff filter of 515 nm. After 15 s recording in basal conditions, 50 µl of graded concentration (10^−12^ to 10^−6^ M) of different peptides (four-fold final concentration) was added to the incubation medium with the built-in eight-channel pipettor at a rate of 62 µl/s to assess their agonistic activity. After subtraction of mean fluorescence background from control wells without Fluo-4 AM, baseline was normalized to 100%, and fluorescence peak values were determined for each concentration of peptide. Potency (EC_50_) and efficacy (*E*_max_) (Table 3) were determined with the Prism 6.0 software (GraphPad Software In., La Jolla, CA, USA) using a four-parameter logistic equation to fit peak fluorescence data.

**Table 3:** Functional parameters of the response of KissR-1 CHO-K1 cells to treatment with eel kisspeptins as measured using FlexStation technology.

Peptide	*E*_max_ ± SEM (%)	EC_50_ ± SEM (nM)
Eel Kp1(10)	235.5 ± 8.4	8.5 ± 4.5
Eel Kp1(15)	276.6 ± 5.3	11.8 ± 2.0
Eel Kp2(10)	259.9 ± 9.5	56.6 ± 27.7
Eel Kp2(12)	255.9 ± 6.7	134.7 ± 18.9

### Tissue Distribution of *Kiss* Transcripts in the European Eel

Various tissues were individually collected from eight freshwater female silver European eels to investigate the distribution of *Kiss1* and *Kiss2* expressions using qPCR. The following tissues were sampled, stored in RNAlater (Ambion-Inc., Austin, TX, USA), and kept frozen at −20°C until RNA extraction: brain, pituitary, eye, liver, kidney, intestine, spleen, muscle, adipose tissue, and ovary. The brain was dissected into six parts olfactory bulb, telencephalon, mesencephalon, diencephalon, *cerebellum*, and *medulla oblongata*. In addition, testes from eight freshwater male silver European eels were also collected.

### Primary Culture of Eel Pituitary Cells and *In Vitro* Treatments

#### Dispersion and Culture

Dispersion and primary culture of pituitary cells from 30 freshwater female silver eels were performed using an enzymatic and mechanical procedure as described by Ref. (33) and as previously used for the test of heterologous kisspeptins (22). Cultures were performed in serum-free culture medium (Medium 199 with Earle’s salt, sodium bicarbonate, 100 U/ml penicillin, 100 µg/ml streptomycin, and 250 ng/ml fungizone) (Gibco, Thermo Fisher Scientific, Villebon sur Yvette, France) at 18°C under 3% CO_2_ and saturated humidity.

#### *In Vitro* Treatments

Hormonal treatments were started 24 h after the beginning of culture to allow cell attachment (Day 0). Replicates of five wells for control and each treated group were used. Eel Kp1(10), Kp1(15), Kp2(10), and Kp2(12) were tested (see Table 2). Kisspeptin stock solutions (10^−4^ M) were prepared in ultrapure water and stored at −20°C. Stock solutions were diluted in culture medium just before addition to the culture wells. Culture medium was changed and kisspeptins were added to the cells on Day 0, Day 3, and Day 7. Control wells were treated with similar dilutions of ultrapure water. Cultures were stopped on Day 10, according to Ref. (22). The effects of treatments (10^−7^ to 10^−11^ M) were tested in at least three independent experiments performed on different cell preparations from different batches of fish.

### RNA Extraction and cDNA Synthesis

Tissue samples were individually homogenized by sonication in Trizol, and total RNAs were extracted according to the manufacturer’s instructions (Invitrogen). Following extraction, samples were treated with DNase I (Roche, Meylan, France), and the first strand of cDNA was synthesized from 400 ng of total RNA using Superscript III reverse transcriptase (Invitrogen) and random hexamer primers. The reaction was performed according to the following thermal conditions with an initial step at 25°C for 10 min followed by incubation at 50°C for 60 min and 70°C for 15 min. The samples obtained were stored at −20°C until qPCR. The extracted RNAs were the same as in Ref. (19).

For cell cultures, total RNA was directly extracted in wells using the Cell-to-cDNA II Kit (Ambion, Thermo Fisher Scientific) according to the manufacturer’s recommendations. Cells were washed with sterile phosphate buffer (Gibco) and lysed with Cell Lysis II Buffer (80 µl/well). The lysates were digested with RNase-free DNase I (Roche). Four microliters of RNA solution of each sample was then reverse transcribed with a SuperScript III First Strand cDNA Synthesis Kit (Invitrogen). The samples obtained were stored at −20°C until qPCR.

### Quantitative Real-time PCR

Eel *Kiss1*- and *Kiss2*-specific primers for qPCR (Table 1) were designed based on the European eel cDNA sequences cloned in this study and using Primer3 Software (Whitehead Institute/Massachusetts Institute of Technology, Boston, MA, USA). To optimize the assays, different annealing temperatures were tested according to the melting temperature (*T*_m_) of primers. To assess their specificity, amplification products were sequenced at GATC Biotech Ltd.

The qPCR primers for European eel β*-actin, lh*β, *fsh*β, *gnrh-r1a, gnrh-r1b*, and *gnrh-r2* were previously designed (25, 27) (Table 1). β*-actin* was used as reference gene. All primers were purchased from Eurofins (Ebersberg, Germany).

Quantitative assays of eel *Kiss1, Kiss2, lh*β, *fsh*β, *gnrh-r1a, gnrh-r1b, gnrh-r2*, and β*-actin* mRNAs were performed using the LightCycler^®^ System (Roche) with SYBR Green I sequence-unspecific detection as previously described (19, 22, 23). The qPCRs were prepared with 4 µl of diluted cDNA template, 2 µl of PCR grade water, 2 µl of SYBR Green master mix, and 1 µl of each forward and reverse primers (0.5 pmol each at final concentration). The qPCRs were performed as follows: an initial step of polymerase activation for 10 min at 95°C; then 41 cycles of 15 s at 95°C for denaturing, 5 s at 60°C for annealing, 10 s at 72°C for primer extension (β*-actin, lh*β, *fsh*β) or 51 cycles of 15 s at 95°C for denaturing, 5 s at 61°C for annealing, 5 s at 72°C for primer extension, 5 s at 83°C to avoid measurement of non-specific annealing (*Kiss1, Kiss2*) or 42 cycles of 10 s at 95°C for denaturing, 7 s at 61°C for annealing, 4 s at 72°C for primer extension (*gnrh-r2*), or 42 cycles of 10 s at 95°C for denaturing, 10 s at 60°C for annealing, 7 s at 72°C for primer extension (*gnrh-r1a, gnrh-r1b*); and a single final extension step of 5 min at 72°C. Each qPCR run contained a non-template control (cDNA was substituted by water) for each primer pair. The efficiency of primers and the specificity of reaction were assessed as previously described (19). Serial dilutions of cDNA pool of brain and pituitary tissues were run in duplicate and used as a common standard curve and also included in each run as a calibrator.

Quantitative real-time PCR efficiencies for *Kiss1* and *Kiss2* primers (calculated by standard dilution curves) were as follows: *Kiss1* 89.84% and *Kiss2* 88.05%. Assay included a melting curve analysis for which all samples displayed a specific single peak.

Normalization of data was performed using total RNA content for the tissue distribution samples, and using β*-actin* mRNA level for the cell culture samples.

### Statistical Analysis

Results are given as mean ± SEM. Non-parametric tests were performed. Mean values were compared by one-way ANOVA Tukey’s multiple comparison test using Instat (GraphPad Software Inc., San Diego, CA, USA).

## Results

### Identification of European Eel Kisspeptins

#### Cloning of European Eel *Kiss1* and *Kiss2* cDNAs

**Figure 1:**
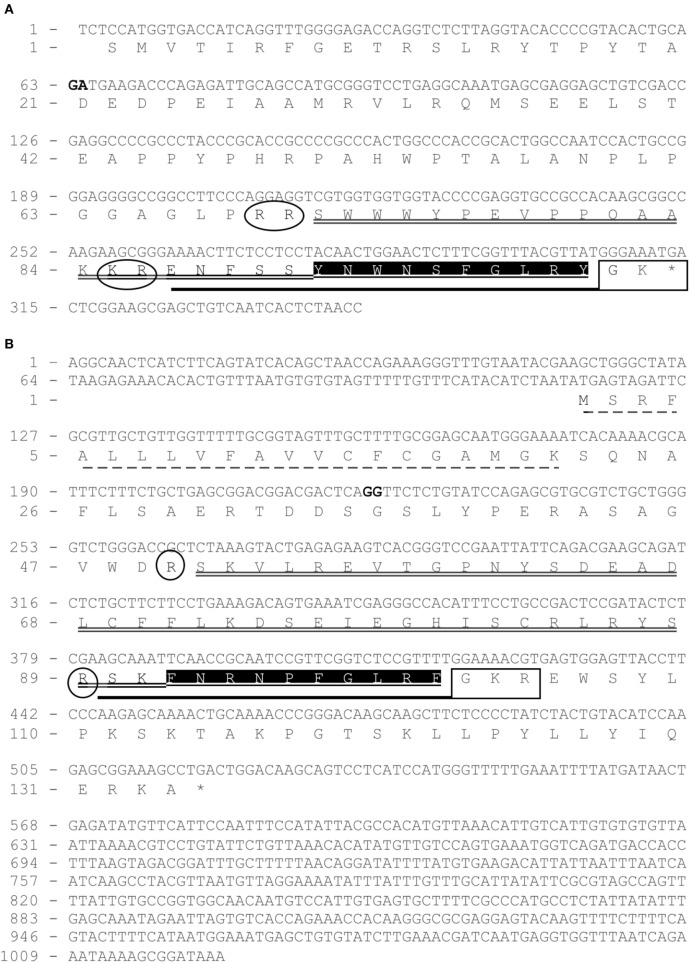
Cloning of eel partial *Kiss1* mRNA **(A)** and complete *Kiss2* mRNA **(B)**. Nucleotides (upper line) are numbered from 5′ to 3′. The exon–intron junctions are indicated by two nucleotides in bold. The deduced amino acids (bottom line) are numbered beginning with the first methionine residue (M) in the ORF (for Kiss2) or with the first codon of the known sequence (for Kiss1 partial mRNA). The asterisk (*) indicates the stop codon, and signal peptide sequence is underlined by a dashed line. Kp1(10) and Kp2(10) are shaded in black. Kp1(15) and Kp2(12) are underlined by a bold line. Kp1(31) and Kp2(51) are underlined by a double line. C-Terminal proteolytic α-amidation sites are boxed in a square and predicted N-terminal cleavage sites are circled.

Using European eel-specific *Kiss1* primers designed on eel *Kiss1*-predicted genomic sequence (23, 26), RACE PCRs, performed on brain cDNAs, led to the cloning of a partial *Kiss1* transcript sequence (EMBL: LT962662) encompassing a partial coding sequence (CDS) of 314 bp and partial 3′-UTRs of 30 bp. Once translated, the cloned *Kiss1* CDS gave a partial 103-aa kisspeptin precursor exhibiting a 10-aa sequence (YNWNSFGLRY) characteristic of the kisspeptin family (Figure 1A).

Using European eel-specific *Kiss2* primers designed on eel *Kiss2*-predicted genomic sequence (23, 26), RACE PCRs, performed on brain cDNAs, led to the isolation of the complete *Kiss2* transcript sequence (EMBL: LT844561). The sequence encompassed 5′- and 3′-UTR of 114 and 495 bp, respectively, and a CDS of 402 bp. Once translated, the cloned *Kiss2* CDS gives a 134-aa kisspeptin precursor exhibiting a 10-aa sequence (FNRNPFGLRF) characteristic of the kisspeptin family (Figure 1B).

BLASTN analyses performed on the European eel draft genome, using the present eel *Kiss1* and *Kiss2* cloned sequences as queries, revealed that each transcript is encoded by two exons. Concerning *Kiss2*, the first exon encoded a 21-aa signal peptide. Both *Kiss1* and *Kiss2* exons-2 encoded the Kiss1 and Kiss2 mature peptides, respectively, including the characteristic 10-aa sequences of the kisspeptin family.

#### Prediction of European Eel Mature Kiss Peptides

The identification of the potential N-terminal and C-terminal cleavage sites of each precursor led to the prediction of two N-terminal extended putative mature peptides: Kp1(15) from *Kiss1* and Kp2(12) from *Kiss2* (Figure 1). Eel Kiss1 presented a conserved dibasic site (KR), 5 amino acids upstream the decapeptide, showing that a mature peptide of 15 amino acids [Kp1(15)] may be produced. Eel Kiss2 possessed a single basic amino acid (R) at position 13, indicating that the *Kiss2* cDNA encoded a putative peptide with 12 amino acids [Kp2(12)]. A 31-aa mature peptide for *Kiss1* and a 51-aa mature peptide for *Kiss2* could also be predicted (Figure 1A).

The sequence of eel Kp1(10) is identical to rat Kp1(10). In contrast, Kp2(10) is a newly identified sequence, which presented at its third position an arginine (R) that possessed different physicochemical properties from amino acids commonly found at this position. Kp1 and Kp2 sequences were followed at their C-terminal side by a proteolytic cleavage and/or an α-amidation motif, i.e., GK motif for *Kiss1* and GKR motif for *Kiss2* precursors, respectively (Figure 1B).

### Tissue Distribution of European Eel *Kiss1* and *Kiss2* mRNAs

**Figure 2:**
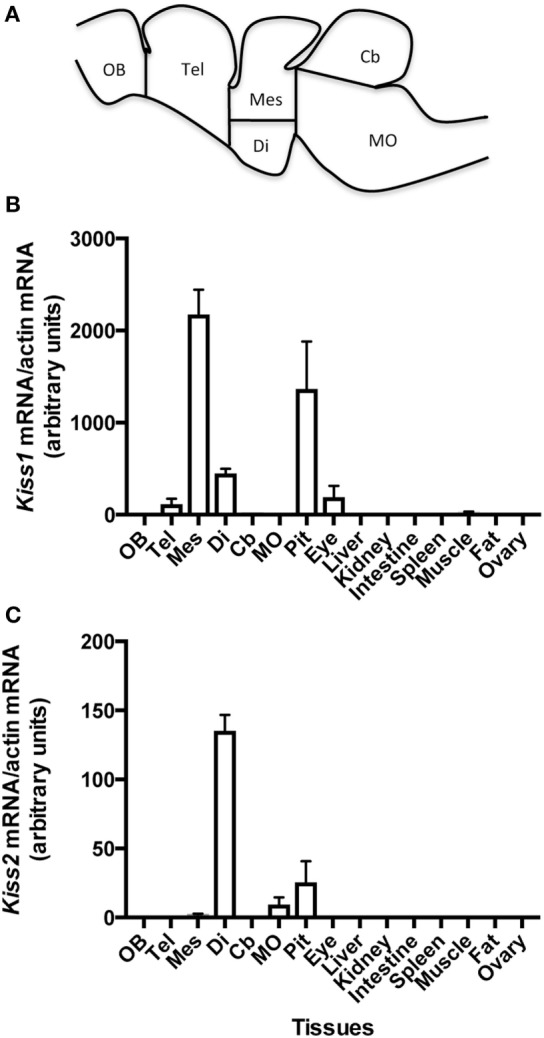
Schematic representation of eel brain with dissection cut sites indicated adapted from Ref. (32) **(A)**, and tissue distribution of the expression of eel *Kiss1* **(B)** and *Kiss2* **(C)** mRNAs. Olfactory bulb (OB), telencephalon (Tel), diencephalon (Di), mesencephalon (Mes), *cerebellum* (Cb), *medulla oblongata* (MO), pituitary (Pit), eye, liver, kidney, spleen, muscle, adipose tissue (Fat), and ovary were dissected from female silver eels. The relative expression of each *Kiss* mRNA was normalized to actin mRNA. Each bar represents mean ± SEM from eight individual samples.

Specific qPCR protocols were developed for each eel *Kiss* and applied to the analysis of their respective tissue distribution (Figure 2).

#### *Kiss1* mRNA Distribution

*Kiss1* mRNAs were mainly expressed in the mesencephalon part of the brain. Its expression was lower in the diencephalon, close to the limit of detection in the telencephalon and *cerebellum* and under detection threshold in the olfactory bulb and *medulla oblongata*. *Kiss1* expression was abundant in the pituitary. In peripheral tissues, low *Kiss1* mRNA levels were measured in the eye and the testis. Its expression was at the limit of detection in muscle and under the detection threshold in the other investigated tissues (liver, kidney, intestine, spleen, adipose tissue, and ovary) (Figure 2B).

#### *Kiss2* mRNA Distribution

*Kiss2* mRNAs were mainly expressed in the diencephalon*. Kiss2* expression was lower in the mesencephalon and in the *medulla oblongata*, and under the detection threshold in the olfactory bulb, telencephalon, and *cerebellum*. *Kiss2* expression was also observed in the pituitary. In peripheral tissues, a low expression of *Kiss2* was detected in the testis. The expression level was under the limit of detection in the other tissues (eye, liver, kidney, intestine, spleen, adipose tissue, and ovary) (Figure 2C).

### Functional Properties of Eel Kiss Peptides

**Figure 3:**
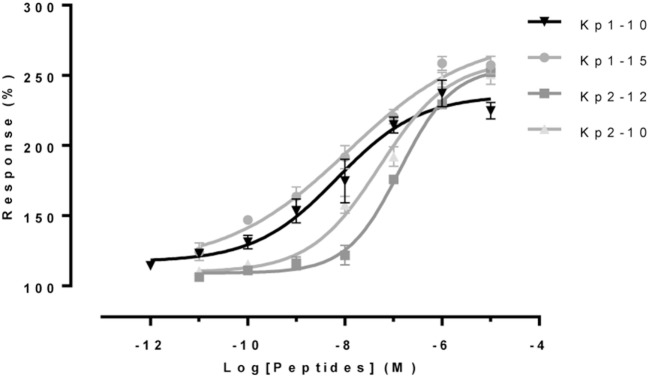
Functional assays of eel Kiss peptides. Levels of [Ca^2+^]_i_ in CHO-K1 cells transfected with rat KissR-1 and stimulated with different doses of eel Kp1(10), Kp2(10), Kp1(15), and Kp2(12) were assessed by using FlexStation technology. After subtraction of mean fluorescence background from control wells without Fluo-4 acetoxymethyl ester, baseline was normalized to 100%, and fluorescence peak values were determined for each concentration of peptide. Values represent mean ± SEM from two independent experiments, and average dose–response curves are shown.

The two 10-aa peptides, Kp1(10) and Kp2(10), and the two predicted mature peptides, Kp1(15) and Kp2(12), were synthesized for functional assays (Table 2). The functional activity of the four synthesized eel kisspeptins was assessed on the kinetics of [Ca^2+^]_i_ in CHO-K1 cells stably transfected with the rat KissR-1, by using a multimode FlexStation III system. All four kisspeptins were able to activate the rat Kiss1R, i.e., to trigger intracellular pathways leading to [Ca^2+^]_i_ increase. As shown in Figure 3 and Table 3, eel/rat Kp1(10) and the other eel Kiss peptides, Kp1(15), Kp2(10), and Kp2(12), displayed the following EC_50_ values: 8.5 ± 4.5, 11.8 ± 2.0, 56.6 ± 27.7, and 134.7 ± 18.9 nM, respectively.

### Effects of Eel Kiss Peptides on Primary Culture of Eel Pituitary Cells

We previously demonstrated that heterologous kisspeptins [human/lamprey Kp1(10); human Kp1(14); lamprey Kp1(13); zebrafish Kp1(10), Kp2(10), Kp1(15), and Kp2(15)] can inhibit *lh*β expression by eel pituitary cells in primary culture, with no effect on *fsh*β (22). In the present study, we tested the effects of concentrations ranging from 10^−11^ to 10^−7^ M of the homologous eel kisspeptins over 10 days in the same culture system.

#### Effects of Eel Kiss Peptides on Gonadotropin Expression

**Figure 4:**
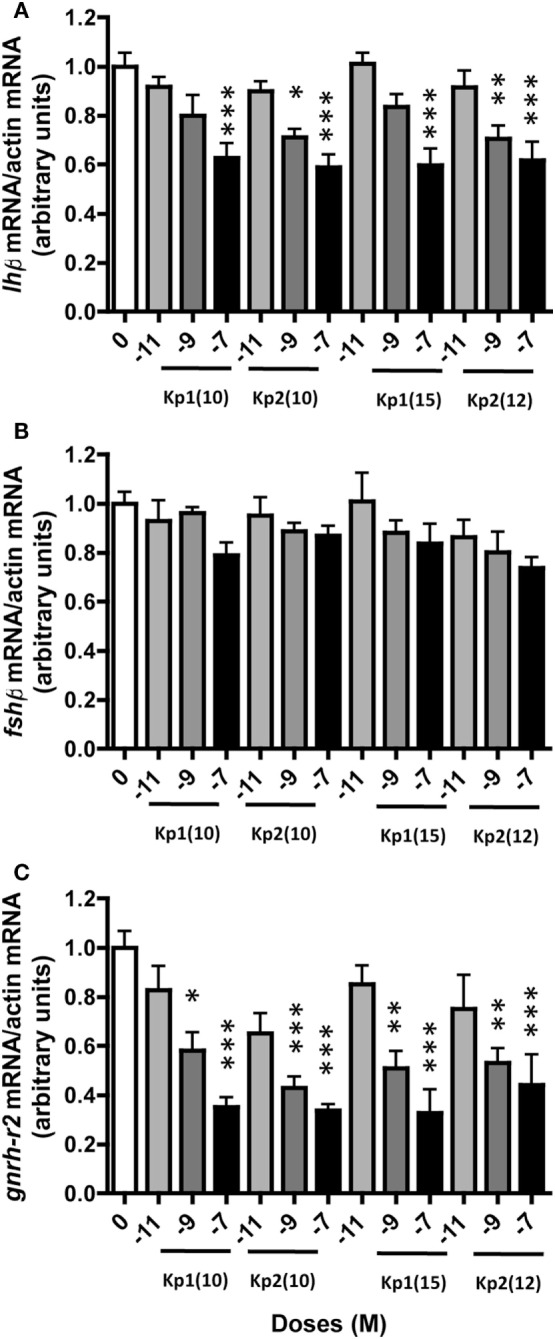
Dose-dependent effect of eel Kiss peptides on pituitary *lh*β **(A)**, *fsh*β **(B)**, and *gnrh-r2* **(C)** expression by eel pituitary cells in primary culture. Pituitary cells were treated with various concentrations (10^−11^, 10^−9^, and 10^−7^ M) of eel Kp1(10), Kp2(10), Kp1(15), and Kp2(12) for 10 days. Pituitary mRNA levels were quantified by quantitative real-time PCR. Data were normalized against β-actin. This figure displays the results of a representative experiment. Each point represents mean ± SEM from five-well replicates. *P < 0.05, **P < 0.01, and ***P < 0.001 *versus* controls, ANOVA.

All four eel synthesized kisspeptins [Kp1(10), Kp1(15), Kp2(10), and Kp2(12)] significantly inhibited *lh*β expression at 10^−7^ M [×0.63 with Kp1(10), *P* < 0.001; ×0.59 with Kp2(10), *P* < 0.001; ×0.60 with Kp1(15), *P* < 0.001; ×0.62 with Kp2(12), *P* < 0.001] (Figure 4A). Inhibition by Kp2(10) and Kp2(12) was also significant at 10^−9^ M (×0.71, *P* < 0.05 and ×0.70, *P* < 0.01, respectively). In contrast, the eel Kiss peptides had no significant effect on *fsh*β expression at any dose tested (Figure 4B).

#### Effects of Eel Kiss Peptides on GnRH Receptor Expression

Among the three eel GnRH receptors, *gnrh-r2* was the only one with detectable specific expression in both control and treated pituitary cells in primary culture; with *gnrh-r1a* and *gnrh-r1b* expressions at the limit of detection, primer dimer peaks (melting curve) were obtained. An inhibitory effect of all four synthesized eel Kiss peptides was observed on the expression of *gnrh-r2*. This inhibitory effect was dose-dependent, the highest inhibition being observed at 10^−7^ M [×0.35 for Kp1(10), *P* < 0.001; ×0.34 for Kp2(10), *P* < 0.001; ×0.33 for Kp1(15), *P* < 0.001; and ×0.44 for Kp2(12), *P* < 0.001] (Figure 4C).

## Discussion

### Eel Kisspeptins

In order to assess the predicted *Kiss1* and *Kiss2* sequences and further investigate the gene exon–intron structures, we performed specific RACE PCR on *Kiss1* and *Kiss2* transcripts. The sequencing of the RACE PCR products and the comparison of their sequences to the European eel genome provided a partial *Kiss1* mRNA encompassing, at least, two exons and a complete *Kiss2* mRNA made of two exons. This structure exhibiting two exons is typical of the conserved structure of *Kiss* genes (34).

Once translated, both *Kiss1* and *Kiss2* transcripts encoded two proteins presenting the characteristics of the kisspeptin precursors. Among those hallmarks, Kiss1 and Kiss2 precursors presented the conserved kisspeptin-10 sequences, i.e., YNWNSFGLRY for Kiss1 and FNRNPFGLRF for Kiss2. Moreover, Kp1(10) and Kp2(10) were followed at their C-terminal extremity by a cleavage and α-amidation signature, i.e., GK and GKR motifs, respectively. For many neuropeptides, α-amidation is essential for biological activity (35, 36). Both eel kisspeptin-10 sequences were encoded by the second exon of each gene transcript in accordance with what has been observed in other species (34).

The eel Kp1(10) sequence was identical to the rat Kp1(10) but, to our knowledge, the only teleost sequence possessing a tryptophan (W) at its third position; other teleosts showed a leucine (L) except chub mackerel with a phenylalanine (F). The eel Kp2(10) sequence was rather unique due to the presence of an arginine (R) at its third position. The occurrence of an arginine at this position has only been reported so far in the Kp1(10) of the musk shrew (*Suncus murinus*) (37). Kp2(10) sequences appeared more conserved among vertebrates than Kp1(10) ones, differing by one amino acid versus three (Figure S1 in Supplementary Material).

Although kisspeptin-10 is considered as the minimal sequence capable of activating KissR (6, 7), *in silico* analysis did not provide any evidence for the existence of mature Kp1(10) or Kp2(10) in any vertebrate including the European eel. Using the NeuroPred tool, we were able to predict the N-terminal cleavage site for each kisspeptin precursor. The predictions of these N-terminal cleavage sites in addition to the C-terminal α-amidation motifs have delineated potential mature peptides that could be directly cleaved from Kiss1 and Kiss2 precursors. Thus, the two likely mature peptides in the European eel were Kp1(15) and Kp2(12). In addition, two potentially longer mature peptides could be predicted: a 31-aa mature peptide for *Kiss1* and a 51-aa mature peptide for *Kiss2*.

The presence of cleavage sites delineating a mature Kp1(15) appears to be conserved among teleosts (Figure S1 in Supplementary Material), except in goldfish *Carassius auratus* (38) and in chub mackerel *Scomber japonicus* (39) in which a Kp1(16) is predicted. In contrast, the presence of a glutamic acid (E) at the first position of the eel mature Kp1(15) seems to be a unique feature among teleosts. Interestingly, the presence of an E residue at the first position of a mature Kp1 is observed in Kp1(16) in the sarcopterygians, including mammals and the coelacanth (*Latimeria chalumnae*), and a non-teleost representative of actinopterygians, a holostean, the spotted gar (*Lepisosteus oculatus*) (23). This feature could thus represent a common characteristic of sarcopterygians and actinopterygians, which has been conserved in the eel and lost in some other teleosts.

The presence of a basic cleavage site leading to a mature Kp2(12) seems to be also conserved among teleosts such as in the European eel (Figure S1 in Supplementary Material). One exception is observed in salmonids (masu salmon and kokanee salmon, *Oncorhynchus nerka*) in which the mature endogenous Kiss2 peptide is a 13-aa sequence (20). The existence of a mature Kp2(12) has been proven in *Xenopus* (21) and in the red-eared slider turtle (20).

### Brain *Kiss1* and *Kiss2* Expression in the European Eel

Both eel *Kiss1* and *Kiss2* mRNAs were mainly expressed in the brain, as shown by specific qPCR, *Kiss1* being more abundant in the mesencephalon and *Kiss2* in the diencephalon. Their expressions were also observed in other parts of the central nervous system (i.e., in the telencephalon and *cerebellum* for *Kiss1* and in the *medulla oblongata* for *Kiss2*), although at lower levels. These distributions, which were obtained in the present study, in females at silver stage may differ in males or in females at a different stage.

In other teleosts presenting two *Kiss* genes, both *Kiss1* and *Kiss2* mRNAs are also expressed in the central nervous system [RT-PCR and qPCR, zebrafish (40–42); RT-PCR, goldfish (43); RT-PCR, sea bass (*Dicentrarchus labrax*), medaka (*Oryzias latipes*) (42); RT-PCR and qPCR, chub mackerel (44); qPCR, rohu, *Labeo rohita* (45); qPCR, *Catla catla* (46, 47); qPCR, pejerrey, *Odontesthes bonariensis* (48)], suggesting that kisspeptins exert important functions in the teleost brain. Anatomical studies, using ISH and laser-capture microdissection coupled with qPCR, have demonstrated that each transcript is located in different brain nuclei: *Kiss1* mRNA is observed in the ventral *habenula*, while *Kiss2* mRNA is found in the preoptic region and in the hypothalamus [for review see Ref. (49); European sea bass (50)]. Nevertheless, in striped bass *Morone saxatilis* (51) and in chub mackerel (39), no *Kiss1* expression was reported in the habenular nucleus. In zebrafish, generation of specific antibodies for each kisspeptin type made it possible to evidence that only Kiss2 neurons send projections to GnRH neurons and pituitary, suggesting a prominent involvement of Kiss2 rather than Kiss1 in the regulation of the gonadotropic activity of this species (52). This is probably also the case in the eel, as *Kiss2* is more actively expressed than *Kiss 1* in the diencephalon, which is the main neuroendocrine region of the brain.

In a previous study, we showed the expression of the three eel Kiss receptors in the brain (19). These receptors are differentially expressed in various brain regions. *KissR-1* mRNAs is widely expressed in all parts of the eel brain. This receptor is the unique receptor present in placental (eutherian) mammals; the European eel is the only extant teleost shown so far to possess KissR-1, which seems to have been lost in other teleosts (19, 23, 24). Eel *KissR-2* mRNAs is mainly expressed in the telencephalon and the di-/mesencephalon, while *KissR-3* expression is primarily observed in the di-/mesencephalon. These data suggest potential multiple actions of kisspeptins in the eel brain that need further investigations.

### Pituitary *Kiss1* and *Kiss2* Expression in the European Eel

Both *Kiss1* and *Kiss2* transcripts were expressed in the eel pituitary. In other teleost species possessing two kisspeptin genes, different observations have been reported. Only *Kiss2* expression is detected in zebrafish (52) and pejerrey (48) pituitaries, while only *Kiss1* is expressed in the chub mackerel pituitary (44). In the medaka, none of the two kisspeptin genes is expressed in the pituitary (53), while in the sea bass, both genes are expressed (54), as in the eel.

We previously showed that *KissR-1* and *KissR-2* are the two receptors expressed in the eel pituitary (19). These data suggest that, in the eel, the pituitary could be a target for the neuroendocrine action of both cerebral and locally produced pituitary kisspeptins. Similarly, pituitary expression of *Kiss* and *KissR* mRNA, as well as the presence of their respective proteins, has been observed in mammals and amphibians [human (6, 7); rat (55–57); ovine (58); and amphibians (21, 59)]. The occurrence of kisspeptin and its receptor in the pituitary supports the notion that the peptide may exert paracrine and/or autocrine actions in this tissue.

### Peripheral *Kiss1* and *Kiss2* Expression in the European Eel

In the eel, *Kiss1* and *Kiss2* were weakly expressed in the testis (data not shown) and not in the ovary at the silver stage (prepubertal blockade), which cannot predict a higher expression at a more advanced sexual stage. In other teleosts possessing two kisspeptin genes, different situations have been reported. In some species, both kisspeptins are detected in the gonads [medaka and zebrafish (42, 53); sea bass (60); rohu (45); and *Catla catla* (46, 47)]. In contrast, in the chub mackerel, *Kiss1* is expressed in the gonads of both sexes, while *Kiss2* expression is not detected (44), whereas in the pejerrey, expression of both genes is very low in the gonads (48). We previously showed a strong expression of *KissR-1* in both the ovary and testis of the European eel (19), which supports a potential endocrine action of *Kiss1* and/or *Kiss2* on European eel gonads.

In other peripheral organs, which do not belong to the brain–pituitary–gonad axis (muscle, liver, fat, kidney, intestine, spleen, and eye), eel *Kiss1* and *Kiss2* expression levels were under the detection limit except in the eye, where *Kiss1* mRNA, but not *Kiss2*, was clearly expressed. We previously showed the expression of *KissR-1* in the eye, suggesting the potential involvement of the kisspeptin system in the local regulation of visual functions. *Kiss1* and *Kiss2* expression also occurs in the eye of other teleost species [zebrafish and medaka (42); rohu (45); and pejerrey (48)]. Kisspeptin receptor mRNAs are also observed in the eye of zebrafish [*KissR-3* only (61)] and pejerrey [*KissR-2* and *KissR-3* (48)]. Interestingly, in the medaka, eye development is interrupted after zygotic knockdown of *Kiss1* (62), implying a possible function of the kisspeptin system during retina ontogenesis. Few data are available in other vertebrates. In X*enopus*, expression of *Kiss-1a* and *Kiss-1b* is observed in the eye, while none of the three kisspeptin receptors and neither *Kiss-2* are expressed in this organ (59). In mammals, one study supports a role of kisspeptin as metastasis suppressor gene in the eye, as the expression of both *Kiss1* and its receptor is detected in human uveal melanoma cell lines and correlates with survival rate (63).

### Functional Properties of Eel Kiss Peptides

We demonstrated that all four synthesized eel Kiss peptides were able to bind kisspeptin receptor in heterologous system (CHO-K1 cells stably transfected with rat KissR-1), inducing a rise in intracellular calcium. Eel Kp1(15) exhibited about the same potency to activate rat KissR-1 than eel Kp1(10), which is identical to rat Kp1(10). Interestingly, eel Kiss2-derived peptides were also able to activate rat KissR-1, as zebrafish (59) and sea bass (60) peptides did with human KissR-1.

Zebrafish Kiss peptides are also highly efficient for activating mammalian (human) KissR-1 in COS-7 cells [zebrafish Kp1(10) (41)] and in CV-1 cells [zebrafish Kp1(15), Kp2(10), and Kp2(12) (59)]. Similarly, in CHO cells, sea bass Kiss peptides [sea bass Kp1(10), Kp2(10), Kp1(15), and Kp2(12)] can efficiently activate human KissR-1, while, in the case of mouse KissR-1, sea bass Kp2(10) is not able to induce any activation (60).

Teleost models, other than eel, possess only two receptors, homologous to eel KissR-2 and eel KissR-3, respectively, as they have lost their KissR-1 paralog, homologous to eel and human KissR-1 (19). For clarity, the nomenclature of Pasquier et al (19, 24) for KissR will be used in the following paragraphs. In teleost models with two receptors, various studies have shown differential affinities of Kiss peptides toward homologous receptors. In zebrafish [CV-1 cells (59)] and sea bass [CHO cells (60)], Kp2(12) exhibits higher potency for activating KissR-3, while Kp1(15) exhibits a preference for KissR-2. These data are in agreement with the neuroanatomical distribution of Kiss and KissR-expressing neurons in these two species [zebrafish (52); sea bass (50, 64)]. However, contradictory results have been obtained by other authors in zebrafish, KissR-2 being activated by both Kp1(10) and Kp2(10), while KissR-3 is preferentially activated by Kp1(10) [COS-7, CHO-K1, and HEK293 cells (61)]. In striped bass, Kp1(15) and Kp2(12) activate KissR-3 with the same potency, while KissR-2 is more efficiently activated by Kp2(12) [COS-7 cells (65)]. In the chub mackerel, the predicted mature peptide Kp1(16) is more active than the shorter Kp1(15) on KissR-3 (39).

Furthermore, in these homologous receptor and peptide systems, distinct intracellular signal transduction pathways can be activated. KissR-2 and KissR-3 signals can be transduced *via* both PKA and PKC pathways in zebrafish (41), medaka (66), chub mackerel (67), and sea bass (60). In goldfish, the PKA pathway is activated by Kiss1/KissR-3, while PKC pathway is induced by Kiss2/KissR-2 (43). In the southern Bluefin tuna (*Thunnus maccoyii*) and in the yellowtail kingfish (*Seriola lalandi*), KissR-2 (the only receptor present in these species) shows stronger transduction *via* the PKC than the PKA pathway (68), while in the orange-spotted grouper, *Epinephelus coioides*, no PKA could be activated (69). In mammals (6, 7) and in the bullfrog *Rana catesbeiana* (21), KissR-1 conveys its signal *via* the PKC pathway and not the PKA pathway.

As, in the eel, three kisspeptin receptors, KissR-1, KissR-2, and KissR-3, are present, future studies should aim at investigating the potency of homologous kiss peptides to activate each eel KissR and study their signal transduction pathways.

### Biological *In Vitro* Activities of Eel Kiss Peptides on Eel Pituitary Cells

All four synthetic eel kisspeptins specifically and dose-dependently inhibited *lh*β expression by eel pituitary cells in culture, while they had no effect on *fsh*β transcripts. These data confirm, using homologous peptides, the specific inhibitory effect of kisspeptin on *lh*β in the European eel that we previously reported with heterologous Kiss peptides (22).

This paradoxical inhibitory effect contrasts with the general action of kisspeptin as an activator of puberty and reproduction, mostly at the brain level (70), but also directly on the pituitary [for reviews see Ref. (71, 72)]. Our results in the European eel suggest that kisspeptins encoded by both *Kiss1* and *Kiss2* have an inhibitory *in vitro* effect on *lh*β expression. Another study, in the striped bass, reported that Kp1(15) had an inhibitory effect on *lh* expression, while Kp2(12) stimulated LH release (65). In contrast, in other studied teleosts, the action of kisspeptin is generally either stimulatory or absent as in mammals. For instance, in goldfish, homologous Kp1(10) and Kp2(10) are inactive on LH release by pituitary cells from sexually mature females (43), whereas homologous Kp1(10) increases the release and expression of LH by pituitary cells from mixed sexes at late stages of sexual regression (38). In the sea bass, Kp2(12) induces both *lh* expression and LH release by pituitary cells obtained from mature males, while Kp1(15) has no effect (73). In (striped and sea) basses, the different actions of Kiss1 and Kiss2 on LH regulation observed *in vitro* have also been reported *in vivo*. In the sea bass, Kp2(10) is significantly more potent than Kp1(10) in inducing LH secretion after intramuscular (im) injection to both prepubertal and adult fish (42). In the striped bass, im injection of Kp1(15) and Kp2(12) induces plasma LH levels in a stage-dependent manner: Kp1(15) has no effect on LH at pubertal stage, while both peptides could increase LH levels at gonadal recrudescence (51). Our results in the European eel suggest that kisspeptins encoded by both *Kiss1* and *Kiss2* have no *in vitro* effect on *fsh*β expression. In a few studies, a stimulatory effect of kisspeptins has been reported on FSH. In the striped bass, Kp1(15) and Kp2(12) stimulate FSH at both the gene transcription and peptide secretion levels (65). In the sea bass, Kp2(12), but not Kp1(15), can induce *in vitro* FSH release (73). Recently, it was reported that, in amphioxus (*Branchiostoma japonicum*), injection of amphioxus kisspeptin-like could upregulate the expression of *gpb5*, a paralog of glycoprotein vertebrate-like β subunit (74). Our results on primary cultures of eel pituitary cells indicate that Kiss peptides may act directly at the pituitary cell levels in the eel. The action of Kiss peptides may be exerted directly on pituitary gonadotrophs or *via* other pituitary cells. We have already shown that heterologous Kiss peptides did not change the expression of *gp*α, *gh, fsh*β, and *tsh*β in the same *in vitro* system (22), but indirect action on LH cells may occur *via* some other factors produced by pituitary cells other than LH cells. Our previous studies revealed that Kiss receptors (KissR-1 and KissR-2) are expressed in the eel pituitary (19). Future *in situ hybridization* studies would be necessary to decipher whether KissR and which type(s) are expressed by LH cells. Future experiments may also aim at investigating the *in vitro* effects of specific antagonists for KissR on *lh*β and *gnrh-r2* expression. In mammals, including humans, Kiss peptides may act not only *via* their cognate receptor (Kiss-R = GPR54) but also *via* other RF-amide receptors that show less specificity such as NPFF receptors 1 and 2 [for instance see Ref. (75)]. This opens further research avenues aiming at characterizing the full complement of RF-amide receptors in the eel.

Besides the inhibitory action on *lh*β expression of eel kisspeptins, the present study reveals a dose-dependent inhibitory effect on the expression of *gnrh-r2* by primary culture of eel pituitary cells. This receptor is the one increased during experimental maturation in both female and male eels (25). To the best of our knowledge, only a single other study investigated the effect of kisspeptin on GnRH receptor in teleost. In female striped bass at dummy run phase (ovarian development is initiated but not completed), chronic administration *in vivo* of both Kp1(15) and Kp2(12) induces a decrease of pituitary *gnrh-r* mRNAs (76). In other vertebrates, the few studies available have been mainly performed in mammals and demonstrate a stimulatory or an absence of effect of kisspeptins on the expression of GnRH receptor. Using the mouse pituitary gonadotroph LβT2 cell line, Witham and collaborators (77) have found that kisspeptin treatment cannot activate GnRH receptor promoter, but, in contrast, Mijiddorj et al. (78) have recently demonstrated that kisspeptin increases the expression of GnRH receptor. While the presence of Kiss/KissR is under question in birds, repeated injections of human Kp1(10) upregulated pituitary expression of type II (but not type I) *gnrh-r* in the juvenile female Japanese quail (*Coturnix japonica*) (79). In the anuran amphibian *Pelophylax esculentus*, Kp10 treatment of testes in culture upregulated the expression of the three *gnrh-r* before and during the reproductive periods, and this effect was completely abolished/counteracted by the antagonist Kp-234 (80).

Our finding suggests that, in the eel, kisspeptins decreased *lh*β expression directly at the pituitary level and also decreased pituitary sensitivity to GnRH by downregulating GnRH receptor expression, leading to a double inhibitory control. The kisspeptin system may thus contribute to the strong inhibitory control of puberty observed in the European eel. This inhibition in the eel, which contrasts with the stimulatory role of kisspeptin in the reproduction of other vertebrates, reveals evolutionary change in the reproductive role of kisspeptin.

## Author Contributions

JP, A-GL, and FD: cloning—qPCR (tissue distribution). JP and KR: primary cultures. BL and JL: synthesis of kiss peptides. CD, AM-H, and JL: binding studies. JP, HV, JL, SD, and KR: design—writing. All authors: final approval.

## Conflict of Interest Statement

The authors declare that the research was conducted in the absence of any commercial or financial relationships that could be construed as a potential conflict of interest. The reviewer JA declared a past coauthorship with several of the authors SD and A-GL to the handling editor.
