# Supplementary material for: Eel Kisspeptins: Identification, Functional Activity, and Inhibition on both Pituitary LH and GnRH Receptor Expression
Source: Front Endocrinol (Lausanne). 2018 Jan 8;8:353. doi: 10.3389/fendo.2017.00353 (PMC5766898; doi:10.3389/fendo.2017.00353)
Supplement: Supplementary file 1 [file Table_1.DOCX]

**Supplemental Figure :** Predicted Kiss peptides in vertebrates. Identical amino acids are shaded in gray.

**Kp1(10)**

Human Kp1(10) YNWNSFGLRF

Rat Kp1(10) YNWNSFGLRY

Musk shrew Kp1(10) YNRNSFGLRY

Xenopus Kp1(10) YNWNSFGLRY

Coelacanth Kp1(10) YNWNTFGLRY

Spotted gar Kp1(10) YNWNSFGLRY

European eel Kp1(10) YNWNSFGLRY

Zebrafish Kp1(10) YNLNSFGLRY

Goldfish Kp1(10) YNLNSFGLRY

Medaka Kp1(10) YNLNSFGLRY

Sea bass Kp1(10) YNLNSFGLRY

Striped bass Kp1(10) YNLNSFGLRY

Catla catla Kp1(10) YNLNSFGLRY

Rohu Kp1(10) YNLNSFGLRY

Pejerrey Kp1(10) YNLNSFGLRY

Chub mackerel Kp1(10) YNFNSFGLRY

**Kp2(10)**

Xenopus Kp2(10) FNFNPFGLRF

Grass lizard Kp2(10) FNFNPFGLRF

Red-eared slider Kp2(10) FNFNPFGLRF

Coelacanth Kp2(10) FNFNPFGLRF

Spotted gar Kp2(10) FNFNPFGLRF

Chub mackerel Kp2(10) FNFNPFGLRF

Sea bass Kp2(10) FNFNPFGLRF

Striped bass Kp2(10) FNFNPFGLRF

Masu salmon Kp2(10) FNFNPFGLRF

Medaka Kp2(10) FNYNPFGLRF

Goldfish Kp2(10) FNYNPFGLRF

Catla catla Kp2(10) FNYNPFGLRF

Rohu Kp2(10) FNYNPFGLRF

Pejerrey Kp2(10) FNYNPFGLRF

Zebrafish Kp2(10) FNYNPFGLRF

European eel Kp2(10) FNRNPFGLRF

Kokanee salmon Kp2(10) FNVNPFGLRF

**Longer Kp1 peptides**

Human Kp1(16) EKDLPNYNWNSFGLRF

Rat Kp1(16) EKDMSAYNWNSFGLRY

Musk shrew Kp1(16) EKEPEAYNRNSFGLRY

Xenopus Kp1(16) EKDLSTYNWNSFGLRY

Coelacanth Kp1(16) ENDLSSYNWNTFGLRY

Spotted gar Kp1(16) EKNLSAYNWNSFGLRY

Chub mackerel Kp1(16) HQDMSSYNFNSFGLRY

Goldfish Kp1(16) KQKVAYYNLNSFGLRY

European eel Kp1(15) ENFSSYNWNSFGLRY

Medaka Kp1(15) QDLSSYNLNSFGLRY

Zebrafish Kp1(15) QNVAYYNLNSFGLRY

Catla catla Kp1(15) QNVAYYNLNSFGLRY

Rohu Kp1(15) QNVAYYNLNSFGLRY

Sea bass Kp1(15) QDVSSYNLNSFGLRY

Striped bass Kp1(15) QDVSSYNLNSFGLRY

Pejerrey Kp1(15) QDVSSYNLNSFGLRY

**Longer Kp2 peptides**

Xenopus Kp2(12) SKFNFNPFGLRF

Grass lizard Kp2(12) SKFNFNPFGLRF

Red-eared slider Kp2(12) SKFNFNPFGLRF

Coelacanth Kp2(12) SKFNFNPFGLRF

European eel Kp2(12) SKFNRNPFGLRF

Chub mackerel Kp2(12) SNFNFNPFGLRF

Sea bass Kp2(12) SKFNFNPFGLRF

Striped bass Kp2(12) SKFNFNPFGLRF

Spotted gar Kp2(12) SKFNFNPFGLRF

Medaka Kp2(12) SKFNYNPFGLRF

Zebrafish Kp2(12 SKFNYNPFGLRF

Goldfish Kp2(12) SKFNYNPFGLRF

Catla catla Kp2(12) SKFNYNPFGLRF

Rohu Kp2(12) SKFNYNPFGLRF

Pejerrey Kp2(12) SKFNYNPFGLRF

Masu salmon Kp2(13) TSKFNFNPFGLRF

Kokanee salmon Kp2(13) TSKFNVNPFGLRF
